# Endolysosomal dysfunction and exosome secretion: implications for neurodegenerative disorders

**DOI:** 10.15698/cst2018.05.136

**Published:** 2018-05-04

**Authors:** André M. Miranda, Gilbert Di Paolo

**Affiliations:** 1 Department of Pathology and Cell Biology, Columbia University Medical Center, New York City, NY 10032, USA.; 2 Taub Institute for Research on Alzheimer’s disease and the Aging Brain, Columbia University Medical Center, New York City, NY 10032, USA.; 3 Life and Health Sciences Research Institute (ICVS), School of Medicine, University of Minho, Braga 4710-057, Portugal.; 4 ICVS/3B's, PT Government Associate Laboratory, Braga/Guimarães 4710-057, Portugal.; 5 Present address: Denali Therapeutics Inc, South San Francisco, CA 94080, USA.

**Keywords:** extracellular vesicles, endomembrane damage, galectin, phosphoinositide, phosphatidylinositol-3-phosphate, lysobisphosphatidic acid, LBPA, sphingolipids, phospholipids

## Abstract

Growing evidence suggests that endolysosomal and autophagic defects are key pathogenic processes in various neurodegenerative disorders, including Alzheimer's disease (AD), Parkinson’s disease (PD), frontotemporal dementia (FTD) and amyotrophic lateral sclerosis (ALS). The causal relationship between these defects and neurodegeneration is supported by human genetic studies identifying disease mutations in genes controlling endolysosomal function and autophagy. The canonical view is that defects in these processes lead to impaired lysosomal clearance of proteins prone to form toxic oligomeric assemblies and/or aggregates, ultimately resulting in cellular pathologies that define these disorders. Because lysosomes mediate the clearance of a large number of lipids, lipid storage is frequently associated with compromised endolysosomal and autophagic function. However, an emerging notion, supported by our recent study on class III phosphatidylinositol 3-kinase (PI3K) Vps34, is that neuronal endolysosomal and autophagic dysfunction can manifest itself with the occurrence of physically damaged endomembranes and with the release of exosomes enriched for Amyloid Precursor Protein COOH-terminal fragments (APP-CTFs) as well as atypical phospholipid bis(monoacylglycero)phosphate (BMP). Here, we summarize our recent findings and their potential implications in the context of lysosomal biology, lipid signaling and neurodegenerative diseases.

A master regulator of both endolysosomal function and autophagy is Vps34 and its lipid product phosphatidylinositol-3-phosphate (PI3P) through which the lipid kinase exerts most of its biological actions in eukaryotic cells. A host of PI3P effectors have been characterized and shown to mediate processes as diverse as endosomal fusion, retrograde transport, sorting of cargoes into intraluminal vesicles (ILVs) of multivesicular endosomes as well as autophagosome biogenesis and maturation. Interfering with the signaling of PI3P and its metabolite, PI(3,5)P_2_ has been implicated in neurodegeneration, as PI3P deficiency was reported in the brain of AD patients and the pathway was genetically linked to ALS and PD. Accordingly, genetic disruption of the Vps34-encoding gene (*Pik3c3*) in mice leads to major neuronal loss, suggesting that ablation and pharmacological inhibition of Vps34 are appropriate models to study the causal relationship between endolysosomal/autophagic defects and neurodegeneration.

Prompted by our findings showing that PI3P is deficient in the brain of AD patients and mouse models thereof, we investigated the consequences of Vps34 kinase inhibition or genetic ablation on autophagy, endolysosomal function and APP processing. We observed that reducing PI3P levels in primary cortical neurons or the neuronal cell line N2a blocks autophagy initiation, causes an increase in autophagy receptor/substrate p62 and polyubiquitinated protein levels and slows degradation of APP-CTFs, including APP-CTF(, the BACE1-derived substrate processed by ((-secretase into amyloid-( (A(). As APP-CTFs accumulate following Vps34 inhibition, it is reasonable to assume that ((-secretase-mediated processing of these fragments is not efficient enough to downregulate their levels. In fact, A( levels were found to be decreased upon Vps34 inhibition.

Given the impact of Vps34 inhibition on endolysosomal function, we hypothesized it may also impair the metabolism of lipids and specifically, those degraded in lysosomes. Using lipidomics, we found that Vps34 inhibition increases levels of specific sphingolipids, namely ceramides and dihydrosphingolipids, somewhat reminiscent of some lysosome storage disorders (LSDs). While dysregulated ceramide metabolism can trigger cytotoxic signaling cascades, including apoptosis and necroptosis, missorting and accumulation of these sphingolipids in membrane subdomains may destabilize lipid bilayers and cause their permeabilization. Thus, we examined levels and subcellular localization of galectin-3, which, similar to other galectins, is a cytosolic lectin with high affinity for glycan (-galactosides that are enriched in the luminal leaflet of endocytic organelles. Thus, intracellular galectin-3 accumulation in the form of ubiquitin-positive puncta denotes permeabilization of endolysosomal membranes, as others have reported. We observed that inhibition or genetic ablation of Vps34 in primary cortical neurons causes accumulation of galectin-3/ ubiquitin/ p62-positive structures (**Fig. 1**). However, those puncta were also positive for cholesterol/sphingolipid-rich membrane marker flotillin, yet, negative for LAMP-1 (although they lied in close proximity to LAMP-1-positive late endosomes/lysosomes), suggesting that they were organelles of endocytic origin lacking standard markers for those compartments. APP did not appear to concentrate on these organelles either. We speculated that their permeabilization causes loss of specific endolysosomal markers through excessive proteolysis. Importantly, accumulation of p62, ubiquitin and galectin-3 on those physically compromised organelles suggested that they are targeted to the autophagy pathway but not properly cleared by lysophagy (*i.e.*, a selective form of autophagy clearing damaged lysosomes), as Vps34 inhibition blocks autophagy initiation. What mechanisms account for endomembrane damage and what endolysosomal compartments are permeabilized in response to Vps34 inhibition? While we have not answered this question, we hypothesized that altered trafficking along the endolysosomal pathway causes secondary accumulation of sphingolipids and possibly sterols, which, in turn, leads to destabilization and permeabilization of endolysosomal membranes. Alternate explanations include increased oxidative stress or loss of glycocalyx coating of their luminal leaflets through missorting of glycoproteins. It will thus be fundamental to better characterize the identity of these damaged structures, perhaps via immunoisolation combined with proteomic and lipidomic analyses. Future work will address whether endolysosomal membrane permeabilization is a common pathogenic process in neurodegenerative disorders associated with LSDs and whether blocking it confers therapeutic benefits.

**Figure 1:**
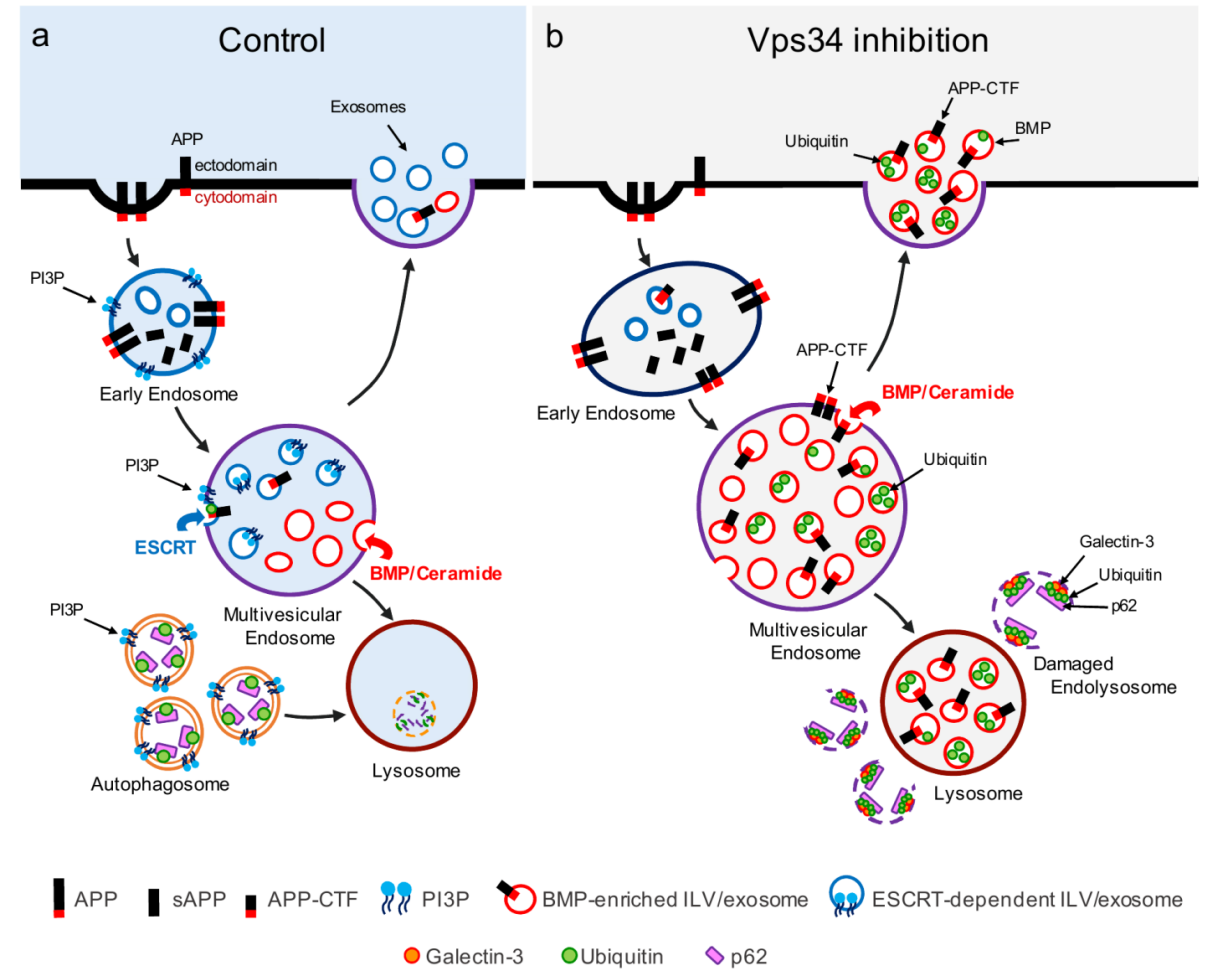
FIGURE 1: Vps34 inhibition causes endolysosomal dysfunction and enhanced release of atypical exosomes harboring poly-ubiquitinated proteins, APP-CTFs and the atypical phospholipid BMP. **(a) **In control cells, newly synthesized APP traffics to the plasma membrane (not shown), where it undergoes clathrin-mediated endocytosis and sorting into PI3P-enriched early endosomes. The acidic endosomal lumen is optimal for amyloidogenic processing by BACE1. APP-CTFs and a pool of full length APP are sorted to intraluminal vesicles (ILVs) of multivesicular endosomes (MVEs) *via* the ESCRT pathway, which requires PI3P and ubiquitination of lysine residues of the APP cytodomain. APP-CTFs can then be(processed by (-secretase into amyloid-( (A(), degraded in lysosomes or secreted in exosomes upon fusion of MVEs with the plasma membrane. Of note, PI3P- and BMP-positive ILVs are segregated from each other within MVEs and, under control conditions, exosomes are largely devoid of BMP. In a separate pathway, autophagosome formation and maturation also requires PI3P and mediate clearance of cytosolic cargoes and damaged organelles labelled with autophagy receptors (*e.g.*, p62) upon their fusion with the endolysosomal system. **(b)** Vps34 inhibition causes enlargement of Rab5-positive early endosomes which are enriched for endosomal adaptor APPL1 instead of PI3P-binding protein, EEA1 (not shown). Through pleiotropic effects on the endolysosomal system, Vps34 inhibition causes accumulation of proteins and lipids in MVEs and lysosomes, resulting in a fraction of them undergoing physical damage, as denoted by the enrichment of galectin-3 on p62- and ubiquitin-positive structures. While PI3P deficiency reduces ESCRT-dependent sorting of cargoes, including APP, into ILVs, local synthesis of ceramide by neutral sphingomyelinase 2 facilitates ILV sorting and secretion of poly-ubiquitinated proteins, APP-CTFs and BMP in exosomes, alleviating lysosomal burden. BMP represents a small fraction (< 5%) of phospholipids measured in total exosome derived from N2a cells treated with the Vps34 inhibitor, compared to more abundant phospholipids, such as phosphatidylcholine (PC) (~30%), phosphatidylserine and phosphatidylethanolamine (PE) plasmalogen (~20% each), PE and ether PC (~10% each), although we predict it may be significantly enriched on a subset of exosomes.

Another key finding reported in our study is that Vps34 inhibition causes robust secretion of exosomes enriched for APP-CTFs (**Fig. 1**). Exosomes are small vesicles secreted upon fusion of multivesicular endolysosomal compartments with the plasma membrane and correspond to the ILVs of those organelles. There is significant heterogeneity in the types of internal vesicles based on their differential protein and lipid composition, and logically, this heterogeneity is carried over in exosomes when internal vesicles are secreted. For instance, it has been reported that internal vesicles can be either PI3P- or BMP-positive, although no studies had reported the presence of either of those lipids in exosomes. Also, whether various subpopulations of exosomes derive from common or distinct endolysosomal compartments remains to be elucidated. We found that by reducing levels of PI3P with the Vps34 inhibitor, the co-secretion of BMP- and APP-CTF-positive exosomes is enhanced, although these molecules may not necessarily reside on the same exosomes (**Fig. 1**). Pharmacological experiments have begun to delineate the mechanisms responsible for this atypical exosome release. First, it is also triggered by inhibition of the V-type ATPase with Bafilomycin A1 (BafA1), suggesting that elevation of endolysosomal pH may be the underlying mechanism, although neither chloroquine nor NH_4_Cl phenocopied these findings, arguing against a simple pH effect (unless BafA1 causes a more sustained increase in endolysosomal pH relative to the other two alkalizers). This may rather reflect an important role of the V-type ATPase in exosome release, as suggested by recent studies. Second, inhibitors of serine palmitoyl transferase and neutral sphingomyelinase 2 both blocked exosomal release of APP-CTFs, suggesting that *de novo* sphingolipid synthesis and ceramide generation are critical. This is consistent with the fact that Vps34 inhibition causes a profound alteration of sphingolipid metabolism, including ceramides. We also note that previous studies from our group have found that the ESCRT pathway, which relies on PI3P primarily due to its ability to recruit ESCRT0/Hrs to endosomal membranes, mediates APP sorting into ILVs and exosome. However, Vps34 inhibition and the ensuing drop in PI3P levels shunt APP/APP-CTFs into an ESCRT-independent pathway, leading to robust exosomal secretion of APP-CTFs. This highlights the plasticity of ILV sorting and exosomal secretion routes for specific cargoes. It remains unclear if BMP is required for secretion of APP-CTF and other endolysosomal cargo in exosomes, a question that may be addressed by identification of the BMP-synthesizing enzymes and their manipulation.

What could be the purpose of exosome release triggered by endolysosomal dysfunction? Both cell-autonomous and non-autonomous aspects must be considered. Growing evidence indicates that exosomes can serve as vectors for eliminating DNA, proteins or lipids. Our work, along with studies from others, suggests that exosome release is a *bona fide* cell-autonomous pathway accounting for the elimination of undesired and/or undigested endolysosomal cargoes, such as toxic proteins or lipids, which cannot be efficiently degraded in lysosomes when the latter are compromised. Concerning APP-CTF(, their exosomal elimination may decrease their inherent cellular toxicity or reduce their processing by (-secretase into A(, which is also deleterious. Similar mechanisms may apply to aggregate-prone proteins such as(tau, (-synuclein ((-syn), and TDP-43, which accumulate primarily in the neurons of patients with AD, PD and ALS/FTD, respectively. In fact, if exosome release is robust enough to eliminate these toxic intracellular proteins, blocking exosomal release may cause their intracellular accumulation and increase their cellular toxicity, perhaps exacerbating intracellular pathologies.

Regarding potential cell non-autonomous functions, growing evidence suggests that exosomes participate in cell-cell communication. In contrast to diffusible, soluble molecules that ensure long-range signaling within a tissue, an organ or an organism, exosomes are more likely to play paracrine roles with limited spreading abilities, unless they reach blood circulation or the cerebrospinal fluid (CSF) or undergo long-range transcytosis along neuronal processes upon internalization. Because exosomes harbor intracellular molecules such as proteins and lipids, the very presence of such molecules on the outer leaflets of their lipid bilayers likely plays a signaling role, driving specificity in cell-cell communication. For instance, the molecules exposed to the surface of exosome could determine what cell types ultimately captures them and transmits their signals, through specific cell surface receptors. In the case of exosomes released upon inducing lysosomal dysfunction in brain tissue, they may carry "eat me signals" destined for immune cells of the brain, such as microglia, which could help eliminating the toxic waste neurons cannot handle. They could also carry danger-associated molecular patterns (DAMPs) stimulating innate immune receptors on astrocytes or microglia, favoring chemotaxis and deleterious gliosis. Future studies will address whether neuronal exosomes triggered by lysosomal dysfunction are endowed with signaling abilities towards glial cells and what the physiological and pathophysiological consequences of this communication are.

In the realm of pathology, exosomes may also contribute to the spreading of neuronal aggregates within the central nervous system, including tau and ((-syn pathologies in AD and PD, respectively. While prion-like modalities have been proposed to mediate the spreading of these protein aggregates, the underlying molecular and cellular mechanisms are still unknown. One of the challenges is that tau and ((-syn must be unconventionally secreted, re-internalized and crossing the endolysosomal membrane in order to nucleate aggregates in the cytoplasm of "host cells". While our study has focused primarily on APP-CTFs, other studies have found that lysosomal dysfunction triggers the exosomal secretion of ((-syn. Exosomal APP-CTFs themselves have been found by Sadoul and colleagues to be processed by the target neurons’ (-secretase after selective binding of exosomes to, and internalization by, their dendrites. Generally, exosomes represent powerful vectors for cell-cell communication, both in physiology and pathophysiology.

Finally, our work has implications for biomarker discovery. Since lysosomal dysfunction causes robust secretion of APP-CTFs and BMP, measuring those molecules in bodily fluids (*e.g.*, plasma or CSF) of patients and preclinical models for disorders associated with lysosomal dysfunction, including AD, PD, ALS, FTD and obviously LSDs, may be informative, and may also provide novel opportunities in clinical settings for identification of pharmacodynamic biomarkers in relation to treatments that improve or correct lysosomal dysfunction.

